# Patient safety education for undergraduate medical students: a systematic review

**DOI:** 10.1186/1472-6920-11-33

**Published:** 2011-06-14

**Authors:** Yanli Nie, Lin Li, Yurong Duan, Peixian Chen, Bruce H Barraclough, Mingming Zhang, Jing Li

**Affiliations:** 1 Chinese Evidence-Based Medicine Centre, West China Hospital Sichuan University, Chengdu 610041. P.R. China; 2 West China School of Medicine, Sichuan University, Chengdu 610041. P.R. China; 3 Royal Australasian College of Surgeons. East Melbourne VIC 3002 Australia; 4 Department of Evidence-Based Medicine and Clinical Epidemiology, West China Hospital, Sichuan University, Chengdu 610041. P.R. China

## Abstract

**Background:**

To reduce harm caused by health care is a global priority. Medical students should be able to recognize unsafe conditions, systematically report errors and near misses, investigate and improve such systems with a thorough understanding of human fallibility, and disclose errors to patients. Incorporating the knowledge of how to do this into the medical student curriculum is an urgent necessity. This paper aims to systematically review the literature about patient safety education for undergraduate medical students in terms of its content, teaching strategies, faculty availability and resources provided so as to identify evidence on how to promote patient safety in the curriculum for medical schools. This paper includes a perspective from the faculty of a medical school, a major hospital and an Evidence Based Medicine Centre in Sichuan Province, China.

**Methods:**

We searched MEDLINE, ERIC, Academic Source Premier(ASP), EMBASE and three Chinese Databases (Chinese Biomedical Literature Database, CBM; China National Knowledge Infrastructure, CNKI; Wangfang Data) from 1980 to Dec. 2009. The pre-specified form of inclusion and exclusion criteria were developed for literature screening. The quality of included studies was assessed using Darcy Reed and Gemma Flores-Mateo criteria. Two reviewers selected the studies, undertook quality assessment, and data extraction independently. Differing opinions were resolved by consensus or with help from the third person.

**Results:**

This was a descriptive study of a total of seven studies that met the selection criteria. There were no relevant Chinese studies to be included. Only one study included patient safety education in the medical curriculum and the remaining studies integrated patient safety into clinical rotations or medical clerkships. Seven studies were of a pre and post study design, of which there was only one controlled study. There was considerable variation in relation to contents, teaching strategies, faculty knowledge and background in patient safety, other resources and outcome evaluation in these reports. The outcomes from including patient safety in the curriculum as measured by medical students' knowledge, skills, and attitudes varied between the studies.

**Conclusions:**

There are only a few relevant published studies on the inclusion of patient safety education into the undergraduate curriculum in medical schools either as a selective course, a lecture program, or by being integrated into the existing curriculum even in developed countries with advanced health and education systems. The integration of patient safety education into the existing curriculum in medical schools internationally, provides significant challenges.

## Background

Health care outcomes have significantly improved with the scientific discoveries of modern medicine. But we also know as a result of studies undertaken in many countries that alongside these benefits are significant risks to patient safety. An extensive literature has been published about the effect of adverse drug reactions and medication errors since the Harvard study in the USA in 1991 first described the extent of harm to patients. Other countries have found similar results, notwithstanding differences in cultures and health systems [1-6]. In order to change the culture of healthcare organizations to one focused on patient safety, medical students should be able to recognize unsafe conditions, systematically report errors and near misses, investigate and improve such systems with a thorough understanding of human fallibility, and disclose errors to patients [7]. They should be taught about human error and the factors influencing adverse events early in their medical education [8]. Incorporating the knowledge of how to do this into the curriculum of medical schools is an urgent necessity [9].

To help medical schools introduce and promote patient safety education, the World Health Organization (WHO) published the "WHO Patient Safety Curriculum Guide for Medical Schools" in 2009 [9]. It focuses on eleven topics, derived from the evidence based, Australian Patient Safety Education Framework" and underwent formal evaluation of its impact during the first 12 months after publication [10,11]. Traditionally, curricula for medical students have focused on 3 major competencies-medical knowledge, technical skills and judgment -clinical decision making. The non-technical and professional competencies such as situational awareness, teamwork and leadership, communication and collaboration, risk management and human factors are not usually explicitly taught or assessed [9]. This WHO guide will enable and encourage medical schools to include patient safety education in the curriculum.

To date, a number of countries have initiated or implemented some patient safety education or training and have undertaken pilot studies on the knowledge and attitudes of undergraduate medical students towards patient safety and medical error [7,8,12-15]. The majority of these studies used, for evaluation, before and after survey processes that involved patient safety education for medical students [7,8,13-16]. On the other hand, some studies on patient safety education focused primarily on health care providers especially, more senior hospital doctors and nurses rather than undergraduate medical students. With the growing recognition of that "medical errors were usually caused by failures of systems, not failures of individuals" [17], it needs to be understood that patient safety education and training as a system property, has a key role to play in achieving the goal of harm minimization. A more fundamental change is required within healthcare curricular, with clear acknowledgement of the importance of creating a patient safety culture at the very beginning of training [18]. As yet, there are relatively few countries and medical colleges that have followed the trend by implementing patient safety education [7,8,13-16]. Although a number of pilot survey studies have been published on patient safety education, no systematic review has been done of the success of this intervention. This research aims to systematically review studies related to the introduction/implementation of a patient safety education curriculum for undergraduate medical students including the curriculum content, teaching strategies, faculty knowledge and background in patient safety, other resources and outcomes. This work aims to understand and promote this knowledge and recommend implementation strategies.

## Methods

### Inclusion criteria

Studies were included if they satisfied all of the following criteria:

#### Study design

Publications were included of either randomized controlled or non-randomized studies including pre/post or descriptive studies reporting the outcomes of patient safety curriculum/training on knowledge, skills, and attitudes of undergraduate medical students.

#### Study subjects

Studies involving undergraduate medical students were included.

#### Interventions

Delivery of concepts, skills, and knowledge and attitude to patient safety within existing curriculum/training.

#### Outcomes

Understanding of teaching strategies, faculty knowledge and background in patient safety, course design and duration. Evaluation was focused on the patient safety curriculum components, improvement of knowledge, skill and attitudes related to patient safety, reported or described in the included studies.

### Exclusion criteria

Studies such as commentaries, personal viewpoints, theoretical and methodology analyses on patient safety were excluded.

### Literature search

We conducted a comprehensive literature search that included MEDLINE, ERIC, Academic Source Premier (ASP), EMBASE and also three Chinese Databases (Chinese Biomedical Literature Database, CBM; China National Knowledge Infrastructure, CNKI; Wangfang Data). Publications from 1980 to 2009 and relevant reference lists of studies identified in the electronic searching were retrieved. The search terms were: '*medical errors',' patient safety', 'medical education', 'curriculum', 'teach', 'medical student', 'undergraduate'*.

### Study selection

Two reviewers independently selected studies initially based on title, key words and abstract of the retrieved record. Studies that did not meet the inclusion criteria were discarded during the initial review. When uncertainty existed we retrieved and assessed the full text studies if they were available. Differing opinions were resolved by discussion to reach consensus between the reviewers.

### Quality assessment

Two reviewers independently assessed the quality of all included studies using the 13 item quality criteria of Gemma Flores-Mateo and Darcy Reed [19,20]. We added one item, namely "is the course design assessed?" Items 1-6 of the quality criteria, were used to assess the completeness of study, items 7-12 for scientific quality, and items 13 and 14 for reliability and validity of evaluation instruments. We assessed each item as "Yes" (1 point) or "No" (0 point). Quality scores were calculated and classified as: poor quality (score < 6), moderate quality (score between 6 and 9), high quality (score between 10 and 14).

### Data extraction

Two reviewers extracted data that met the inclusion criteria by independently using a pre-specified extraction form containing the following information: study design, study subjects, teaching strategies and content, outcome measures.

### Data analysis

Meta-analysis of pooled results was performed or calculated if data synthesis was possible, otherwise descriptive analysis was conducted.

## Results

### Study searching and selection (Figure 1)

**Figure 1:**
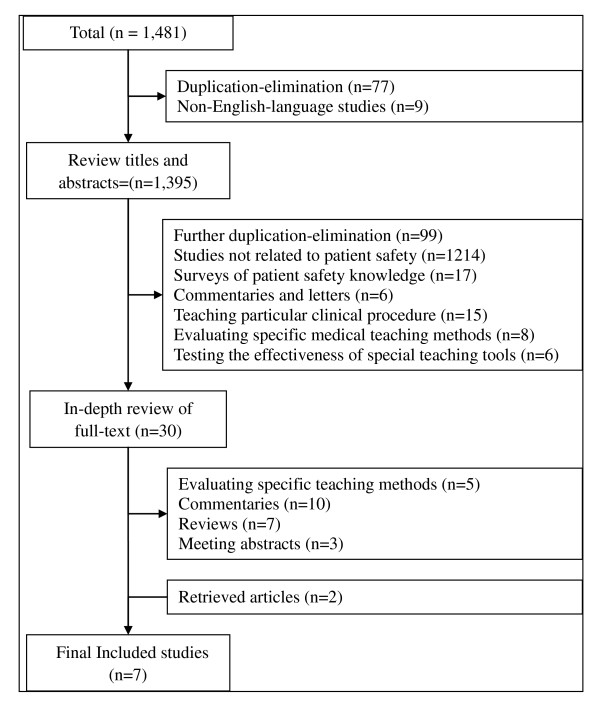
**Flow Diagram for searching and selection processes**.

We identified 1481 studies based on our initial searching. After applying the inclusion and exclusion criteria, a total of seven studies [7,8,12-16] were studied.

### Study characteristics (Table 1)

Table 1 presents some basic information including study design, year of implementation,, characteristics of learner and instructor, teaching content and strategy respectively. Most of the studies were from the USA, and most patient safety courses were implemented in year 3. The teaching faculty were a mixture of interdisciplinary professionals, including clinicians, ethicists and medical education experts. Course design tended to be either as a selective course or incorporated into a clinical rotation of internship training, rather than being integrated into the formal undergraduate medical education system. The duration of the course, course contents and teaching strategies varied across all studies. Most studies were pre and post survey studies of which only one by Anderson 2009 was a controlled study [14]. Three other studies reported the results of a questionnaire and conducted pre-tests [7,8,13].

**Table 1:** Characteristics of included studies

Studies	Study design	Implementation year	School/country	Grade/Number of students	Instructor	Course integrated into	Duration/ frequency	Content								Teaching format							
								A	B	C	D	E	F	G	H	a	b	c	d	e	f	g	h
Halbach 2005	Pre/post survey	2000-01, 2001-02, 2002-03	New York Medical College (USA)	3/572	Standardized patients; Family physicians; Behavioral medicine faculty	Family medicine clerkship	4 hours/1	×		×		×				×	×	×					×
Madigosky 2006	Pre/post survey	2003-2004	University of Missouri-Columbia School of Medicine (USA)	2/92	Volunteer faculty; Ethicists; Medical education experts	Introduction to patient care course	10.5 hours/NR	×	×	×				×	×	×		×		×	×		
Moskowitz 2007	Pre/post survey	2005	Jefferson Medical College (USA)	3/229	Staff in medical education and health care; Clinical faculty in different specialties; High-level directors from four institutes#	Plenary session and workshops	1 day	×	×		×					×			×				
Anderson 2009	Control study Pre/post survey	NR	University of Leicester (UK)	NR/199	Medical education experts	First-aid care course	1 day/9					×		×	×	×	×	×		×		×	
Patey 2009	Pre survey	2004.9-2005.6	Aberdeen Royal Infirmary (UK)	4/110	Anesthetists, physician; industrial psychologist;Clinical psychologist	Core curriculum	5 hours/11	×	×	×			×			×		×		×	×		
Paxton 2009	Pre/post survey	2005.12-2006.12	Henry Ford Hospital (USA)	2+3+4/2+33+1	Surgical residents; Attending staff; Teaching assistants	Clinical rotation	1.5 hours/NR	×	×	×	×		×	×		×				×			
Gunderson 2009	Pre/post survey	2006 spring	University of Illinois at Chicago (USA)	NR/18	Course directors; Observing faculty; Risk-management experts	Optional courses	30 hours/NR					×	×	×		×	×	×		×	×		×

Total				NR/1256				5	4	4	2	3	3	4	2	7	3	5	1	5	3	1	2

NR: Not Report;

# high-level executives from National Patient Safety Agency(NPSA), American Association for the Improvement of Clinical Service, Quality and Safety Research Group, Johns Hopkins University and the dean of the medical school

**Content: **A. medical error, including disclosure/communication and reporting system; B. human factors engineering; C. systems theory/knowledge; D. patient safety regulations/legislation; E. teamwork principles; F. system approach and solutions; G. quality improvement methods, including root cause analysis; H. others

**Teaching formats: **a. interactive lectures/discussion; b. readings; c. case-based learning/discussion; d. seminars; e. small-group discussion; f. role-play; g. interdisciplinary team-working; h. simulation with a standardized patient

### Quality assessment (Table 2)

The quality of included studies was assessed using Darcy Reed and Gemma Flores-Mateo criteria [19,20]. The review found that the lowest score of study quality was 3, while the highest score was 10 (mean = 6.88). All studies met the first six items. But no study conducted power analysis to determine sample size or assessed the reliability of the evaluation questionnaire. Four studies conducted evaluation of course design [7,8,12,14].

**Table 2:** Quality criteria for evaluating studies

Studies	Completeness						Scientific quality of study design						Reliability of evaluation instrument		Score	Grading
			
	1	2	3	4	5	6	7	8	9	10	11	12	13	14		
Halbach 2005	1	1	1	1	1	1	0	0	0	0	1	1	0	0	8	moderate
Madigosky 2006	1	1	1	1	1	1	0	0	0	0	1	1	0	0	8	moderate
Moskowitz 2007	1	1	1	1	1	1	0	0	0	0	0	0	0	0	6	moderate
Anderson 2009	1	1	1	1	1	1	1	1	1	0	1	0	0	0	10	high
Patey 2009	1	1	1	1	1	1	0	0	0	0	1	1	0	0	8	moderate
Paxton 2009	1	1	1	1	1	1	0	0	0	0	0	0	0	0	6	moderate
Gunderson 2009	1	1	1	1	1	1	0	0	0	0	0	0	0	0	6	moderate

1: Is the study purpose easily identified? 2: Are objectives congruent with intervention and evaluation? 3: Is study design appropriate for question? 4: Is study design described in sufficient detail to be replicated? 5: Are teaching methods described in enough detail to replicate? 6: Are statistical tests described? 7: Are raters blinded with respect to group assignment? 8: Is there a similar comparison group? 9: Are confounding variables controlled-for by design or analyses? 10: Has power analysis been conducted to determine sample size? 11: Is the course design assessed? 12: Are long term effects assessed? 13: Is reliability of instruments reported? 14: Is validity of instruments reported? Rating scale: Yes = 1; NO = 0

### Effects on medical students' knowledge, skills, and attitudes about patient safety (Table 3)

All seven studies reported the effects of the patient safety curriculum on students' knowledge, skills and attitudes, and the surveys were all performed by use of a self-made questionnaire [7,8,12-16]. Results are shown in Table 3 and summaries were described as follows:

**Table 3:** Effects of the patient safety on medical students' knowledge, skills, and attitudes

Outcome dimension(s) addressed	Studies	Evaluation design	Quality score	Effects	
				
				Post survey	Follow up
knowledge	Halbach 2005	pre/post survey, follow up	8	↑	↑
	Madigosky 2006	pre/post survey, follow up	8	↑	↑
	Moskowitz 2007	pre/post survey	6	/	/
	Patey 2009	pre survey, follow up	8	/	↑
	Paxton 2009	pre/post survey	6	↑	/
	Gunderson 2009	pre/post survey	6	↑	/
skills	Halbach 2005	pre/post survey, follow up	8	↑	↑
	Madigosky 2006	pre/post survey, follow up	8	Some↑ Some→	Some↑ Some→
	Moskowitz 2007	pre/post survey	6	/	/
	Anderson 2009	pre/post survey	10	↑	/
	Patey 2009	pre survey, follow up	8	/	↑
	Paxton 2009	pre/post survey	6	↑	/
	Gunderson 2009	pre/post survey	6	↑	/
attitudes	Madigosky 2006	pre/post survey, follow up	8	Some↑ Some→ Some*	Some↑ Some→ Some*
	Moskowitz 2007	pre/post survey	6	Some↑ Some→	/
	Patey 2009	pre survey, follow up	8	/	→

	Gunderson 2009	pre/post survey	6	↑	/

↑:improvement or changes in the expected direction

→:no effect or without change or not sustained

/:not report

*:changes in an undesired direction

Knowledge of patient safety included definition and understanding of medical error, rates and types of adverse events in healthcare, error classification, contributing factors to medical error and overview of mechanisms for learning from error. Six studies reported that patient safety knowledge was improved after the course was given [7,8,13-16].

Patient safety skills training included recognition of error, dealing with error, reporting and learning from error, supporting others involved in error.

Students' attitudes to patient safety were explored, focusing on an understanding of a just culture, willingness to learn from mistakes, being prepared to acknowledge and deal with error, being prepared to reflect on practice, and aspects of trust and respect. Three studies evaluating attitudes towards patient safety all reported that attitudes were improved [7,12,16], One study demonstrated there was no change of attitudes after one year follow-up(8).

In addition, three studies evaluated long-term effects of the course [7,8,13]. Four studies surveyed students, the contents and teaching strategies, teaching resources and faculty knowledge and background in patient safety [7,8,13,14]. Six studies obtained full ethical approval from an Institutional Review Board and/or other Ethics Committees [7,8,13-16], two studies were funded by the National Patient Safety Agency [8,14].

## Discussion

The complexity of modern health care increases the risk of error and accidental harm and medical trainees knowledge about patient safety has been shown to be limited [7,8,12-16]. Formal training related to the concepts and principles of patient safety would be expected to address these issues. Although medical schools have begun to incorporate content about patient safety and medical error into their curricula, little has been published so far about these efforts. Our recent initial literature search included MEDLINE, Educational Resources Information Center, Academic Source Premier and EMBASE and Chinese Biomedical Database and China National Knowledge Infrastructure yielded a total of 1481 relevant studies of this subject, with only a few studies meeting the criteria for inclusion. Most are case reports, and none of the studies were identified from China.

### Patient safety in undergraduate education

This review indicates that the addition of patient safety education into the medical school curriculum is most commonly implemented in medical schools in developed countries such as the United States of America and the United Kingdom. Most of these courses are optional courses or are integrated into clinical internship or skill courses, and have not been formally included in the undergraduate medical education system. Also, this literature review shows that there are great difference in course design and contents, who is taught, the teaching resources and faculty development and outcome evaluation. For example, 1) course design and content time ranges from 4 to 30 hours [11,16] and none of studies systematically cover all the accepted key areas of patient safety knowledge. 2) teaching formats: there are eight types of educational format for delivery of the curricula including interactive lectures/discussions, recommended texts, case-based discussions, seminars, small group discussions, role play, interdisciplinary team work and videotaped simulation with a standardized patient; 3) outcome evaluation: all of the included studies adopted a pre/post questionnaire evaluation strategy to measure whether there is an improvement in students' patient safety knowledge, skills and attitudes, but there was no uniform criteria to evaluate the effect of teaching. No study explicitly identified the framework on which the patient safety education curriculum for undergraduate students was based. 4) Student' characteristics also varied from Year 1 to 3 of the medical course.

The results have demonstrated, using pre and post survey methods, that students' knowledge, skills, and attitudes to patient safety improved in most studies [7,11,15,16]. There was also improved implementation of patient safety education in two studies with funding support [8,14] compared to those without funding.

### Quality of included studies

The most commonly inlcuded studies on the teaching patient safety and medical error are descriptive studies, therefore, criteria by Gemma Flores-Mateo et al were used for quality assessement. Seven studies only met the first six items of these quality criteria, while other items were poorly met. As patient safety is a relatively new initiative for medical education, it is assumed that, in future, there will be a growing volume of research in this field with high quality of design.

From four of these studies focused on students knowledge, skills, and attitudes about patient safety [7,8,12,16], the results indicate that the majority of Year 1 medical students reported that they had 'medium low' or 'average' levels of knowledge of error and patient safety issues, but that they hoped to learn more about patient safety knowledge, and skill. To achieve this improvment in knowledge, skills and attitudes, it is unlikely to be sufficient to just construct a system of medical quality assurance and continuous improvement and to build up a harmonious medical environment with a patient safety culture. Addressing the issues from the fundamentals, namely, in undergraduate medical education, is considered to be an important step.

If the implementation of patient safety eduation in medical schools is to achieve the desired outcomes, it should meet local needs in relation to curriculum change and teaching styles and methods and should be based on an evidence based framwork. The "WHO Patient Safety Curriculum Guide for Medical Schools" is an appropriate resource on which to base such activity [9,10]. This curriculum guide aims to prepare students for safer practice in the workplace, inform university faculty about patient safety topics and increase their capacity as patient safety educators, increase the profile of patient safety and encourage international collaboration and research in this field. It also provides a comprehensive curriculum to assist with the teaching and integration of patient safety learning. The guide suggests that a range of teaching methods be used to introduce patient safety topics into the existing curriculum including; the addition of patient safety topics into Problem Based Learning scenarios, the use of high and low fidelity simulation based learning, interactive and didactic lectures, mentoring and coaching, student initiated learning through projects and prescribed activities in the workplace, small group and on line discussion, and the intergration of teaching into practice in the operating room, clinic and at the hospital bedside. It also recognises that there are barriers to be overcome in adding to an already busy curriculum and suggests that university and hospital decision makers and faculty be fully engaged by explaining; the need and rational, and that it can be integrated into the teaching of clinical medicine to enhance existing material; that it does not necessarily need new blocks of time but can be integrated when mapped to the existing curriculum. This process does require recognition of the need for leadership by champions and some developmental resources [9,10].Using this guide and other resources, the Chinese Evidenced Based Medicine Centre, the West China School of Medicine and the West China Hospital, Sichuan University, Chengdu, China, have introduced patient safety education, delivered as selected or continuing education courses, or lectures. The Centre has planned to implemente patient safety education in a number of different educational formats, including, ward round-based teaching, small group learning, case-based discussions, independent study, patient tracking, role play, simulation, as well as incorporation into problem-based learning scenarios. Basic knowledge is integrated into the traditional medical curriculum including the use of a multimedia teaching to deliver real-case-based discussion to teach students about patient safety in the first two years. A greater emphasis is placed on skills training in the later years, such as interdisciplinary activities, professional mentoring and simulation by standardized patients, so that medical students have a full understanding of their healthcare service role in real medical settings [9,10,21-23].

Finally, it is considered that the key to patient safety education lies in prevention, not remedy, so the ideal educational strategy with regard to patient safety is that it should be taught throughout the entire curriculum with all opportunities taken to teach students to prevent mistakes in clinical practice and improve safety and quality of care [13].

## Conclusion

This literature review has shown that there are significant differences in how patient safety education is provided for medical students in relation to course design, and contents, the stage at which it is introduced to the curriculum, evaluation processes and outcomes. in the included studies. While knowledge about the most appropriate way to introduce and teach patient safety into current medical school curricula in order to achieve improvements in care, is still being gathered, the WHO Patient Safety Curriculum Guide for medical Schools is a current evidenced based resource to guide curriculum development and intoduction. In this early developmental stage and with the current knowledge base, patient safety continues to provide major challenges for integration into existing medical education curricula.

## Competing interests

The authors report no conflicts of interest, but note that Mingming Zhang was a member of and Bruce Barraclough the expert clinical lead of the WHO working party for the development of the "WHO Patient Safety Curriculum Guide for Medical Schools". The authors alone are responsible for the content and writing of the article.

## Authors' contributions

MMZ and JL conceptualized and designed the study. LL, YRD, YLN and PXC acquired and analyzed with the support of MMZ. JL provided methodology support. MMZ, LL, YRD and YLN drafted the manuscript. MMZ revised it critically for important intellectual content. Barraclough BH provided expert suggestions and revised the manuscript for English proof. All authors approved the final manuscript for publication.

## Pre-publication history

The pre-publication history for this paper can be accessed here:

http://www.biomedcentral.com/1472-6920/11/33/prepub
